# ‘Excessive sweating is not a feminine thing’: A qualitative study of women’s experiences suffering from primary hyperhidrosis

**DOI:** 10.1371/journal.pone.0254689

**Published:** 2021-07-15

**Authors:** Alexander Shayesteh, Margareta Persson, Christine Brulin, Elisabet Nylander

**Affiliations:** 1 Department of Public Health and Clinical Medicine, Dermatology and Venereology, Umeå University, Umeå, Sweden; 2 Department of Nursing, Umeå University, Umeå, Sweden; De Montfort University, UNITED KINGDOM

## Abstract

**Background:**

Primary hyperhidrosis, excessive focal sweating is a common disease equally affecting men and women. Women tend to seek care more often and assess being more affected by hyperhidrosis in their daily life. The aim of this study was to explore experiences of living with primary hyperhidros in a sample of 15 women.

**Methods:**

Individual, semi-structured interviews with a purposive sample of 15 women diagnosed with primary hyperhidrosis and analysed by qualitative content analysis utilising an inductive approach.

**Results:**

The analysis resulted in a theme, constantly guarding the female image, based on three categories, misfitting the feminine norms, avoiding the attention of others and passing like any woman. Primary hyperhidrosis in women disrupted the ideal feminine appearance. Wearing clothes that concealed hyperhidrosis and distancing from social gatherings, in combination with negative remarks by others, created stress and anxiety and had a negative effect on self-esteem. Women felt poorly understood by others regarding the extent of their sweating and were misunderstood in intimate situations while trying to reduce the sweat production. Choices regarding education and career opportunities were affected, since being exposed and receiving attention due to primary hyperhidrosis was unwanted. Treatment with botulinum toxin liberated women from excessive sweating and removed a social handicap they described living with.

**Conclusion:**

Primary hyperhidrosis in women disrupts the feminine appearance, lowers self-esteem and hinders social interactions. Clinicians assessing primary hyperhidrosis need to be aware that women may report the impairments from primary hyperhidrosis as being more associated with body image and appearance than with functional reductions in daily life. Educating patients, providing accurate information regarding the disease via media and cooperating with patient groups are important for increasing awareness and achieving progress in care for women with primary hyperhidrosis.

## Introduction

Primary hyperhidrosis is characterised by excessive focal and idiopathic sweating [1]. Hyperhidrosis typically starts in adolescence, manifesting itself in areas such as armpits, palms or soles [2]. The disease is equally common in both sexes, and its prevalence has been estimated at 5.5% in Sweden [3] and at 4.8% in the United States [4]. The medical history of patients with hyperhidrosis is usually sufficient for diagnosis, and the Hyperhidrosis Disease Severity Scale (HDSS) gives further guidance on sweating severity and therapy recommendations [5]. HDSS is a 4-point, self-assessment scale with a range from 1 to 4 points in which 3–4 points indicate severe hyperhidrosis [6].

Common treatments for primary hyperhidrosis consist of topical creams containing aluminium and alcohol, which occlude and atrophy the sweat glands, anticholinergic medication and botulinum toxin injections [5]. Surgical removal of the sweat glands could be an option in therapy-resistant cases. An unwanted side-effect associated with surgical procedures is however compensatory hyperhidrosis [1].

In clinical practice, subjective assessment of the severity of hyperhidrosis determines the treatment provided. Although several questionnaires have been developed, international guidelines usually recommend the use of HDSS for this purpose [5]. Gravimetry is seldom used in clinical practice as it is time consuming, and the result could be difficult to interpret. Deficient objective methods for measuring sweat production and lack of consensus regarding excessive sweat production, creates a challenge in diagnosing and providing satisfactory treatment.

Individuals with primary hyperhidrosis seldom seek healthcare [3] and seldom discuss hyperhidrosis with a health care professional as well [2]. Among those who do seek medical attention, women tend to constitute a majority of patients [7]. Deficient knowledge about having a treatable disease, difficulties in finding information, and being afraid of further stigmatization could explain why the majority of affected don’t seek help [8, 9].

As a consequence of hyperhidrosis, negative impacts on mental health and quality of life are reported among affected individuals [8]. The constant need for planning ahead to cope with situations such as shaking hands, speaking in front of others or being obliged to use mandatory clothes, such as school and company uniforms, creates anxiety and stigmatises the individual [8–11]. Hyperhidrosis has also been described influencing choices regarding education and career options [10, 11]. Data regarding the experiences of women living with a stigmatising and visible disease such as hyperhidrosis is scarce. It has been reported that women associate hyperhidrosis with negative self-image and a low self-esteem [8]. In psoriasis, another visible and stigmatising skin disease, women more often than men report stress and anxiety [12], feel discriminated due to stigma [13] and assess lower quality of life despite less severe disease [14]. High importance of physical appearance and the importance of socioeconomic status have been described as possible causes of these discrepancies [15].

Primary hyperhidrosis has formerly been given little attention within the research community, and experiences of women living with hyperhidrosis have, to the best of our knowledge, never been investigated. As there are indications that men and women experience the impairments of hyperhidrosis different, focusing on the burdens of the disease created in women could result in different approaches to management and coping with this chronic disease. Thus, the aim of this study was to explore experiences of women living with hyperhidrosis.

## Materials and methods

### Study design

We conducted semi-structured interviews with a purposive sample of 15 women diagnosed with primary hyperhidrosis. The interviews were analysed by qualitative content analysis utilising an inductive approach.

### Settings and recruitment procedure

Recruitment was done among patients attending the Department of Dermatology and Venereology, Umeå University Hospital, during 2016 and 2017. Inclusion criteria were females with primary hyperhidrosis diagnosed by a general practitioner and verified by a specialist in dermatology–venereology; HDSS score of 3–4, points, indicating severe hyperhidrosis; and ability to express their experiences in Swedish. Women with absent primary sweating site and those who had received treatment with botulinum toxin within 1 year prior to the interview, risking potential recall bias due to the recent treatment, were excluded. A sample of 24 women with appointments due to hyperhidrosis were given oral and written information about the study by AS, and 15 women agreed to participate. Thereafter, suitable times for the interviews were mutually agreed upon. All interviews were performed by AS in a neutral conference room, separated from the clinic and absent of any patient–caregiver associations or consultation. Although those who did not participate were informed that no reason was needed for abstaining, they still offered travelling distances or family situations as causes for not participating. None of the women recruited in this study had received treatment by any of the authors. Participants’ background characteristics are presented in Table 1.

**Table 1:** Characteristics of women participants.

**Women (n)**	15
**Age (range)**	26^a^ (20–53)
**Age at onset (range)**	13^a^ (6–25)
**Occupation (n)**	
Light physical work	13
Heavy physical work	2
**Hyperhidrosis (n)**	
Axillary	7
Palmar	3
Plantar	1
Axillary and palmar	4

^a^ Values given as median years

### Data collection

This study used individual interviews, the most common method for data collection in qualitative healthcare research. Interviews provide data on views, beliefs, motivation and experiences from individuals investigated [16]. An interview guide, “S1 and S2 Protocols”, based on clinical experience and literature review, was constructed to guide the interviews and aid the conversation. The interviews explored the experiences of the informants regarding several areas, such as present time, childhood and adolescence, private and social life, occupation and intimacy. Open-ended questions were used to ‘describe’ or ‘give actual examples’ of situations and thoughts of the participants, while probing questions such as ‘please elaborate’ or ‘could you explain’ were used to clarify certain topics. Verbal vignettes [17] were also used to facilitate shared experiences in sensitive situations. Vignettes covered situations such as the participants’ reactions of shaking hands with others or asking for experiences in intimate situations (Table 2).

**Table 2:** Example of a vignette used during interviews.

You are meeting a new person with your friends. The person reaches out to shake hand with you. You notice that the hand is soaked with sweat. The person with the sweaty hand is a woman. What are your thoughts about the situation? Please, explain Now imagine that the person with the sweaty hand is a man, what are your thoughts about the situation? Please, explain

At the end of the interviews a summary was presented to the participant by AS for further elaboration or clarification. The interviews lasted for 40–50 minutes and were transcribed verbatim by medical secretaries at the same department as the first author.

### Data analysis

The epistemological assumptions in qualitative content analysis are that data should be described with as little interpretation as possible. While data and its interpretation are created by the informant and the researcher, further interpretation during analysis is created by the researcher and the text. Therefore, it is assumed that the text can have several meanings [18].

In this study, an inductive approach of qualitative content analysis was used with the aim of distilling and enhancing the understanding of data [19]. Each meaning unit of transcribed texts was read several times to make sense of the data and to identify content areas according to our aim. The meaning units were condensed by shortening the data material while retaining its core meaning. The condensed meaning units were further labelled with codes describing the content in the unit. Codes sharing similarities defining a specific topic were classified into subcategories. Following a higher logical order, subcategories were abstracted into categories; thereafter, the meaning in the categories was interpreted, resulting in a theme presented as a metaphor [18] that captured the underlying meaning of women’s narrative experiences of hyperhidrosis.

### Rigour

This study considered the four areas in qualitative research: credibility, transferability, dependability and confirmability, suggested by Guba [20]. Qualitative content analysis is a reflective and non-linear process influenced by the researcher’s own pre-understanding and creativity [21, 22]. Working and reworking the data required the first author to engage in reflective thinking by discussing analysis and results, as a continuous process, to reach consensus with other members of the research group. In addition, interviewing 15 informants and achieving saturation in data, ensuring honesty in informants, having frequent debriefing sessions between AS and the research group, providing a dense description of the findings and having experienced researchers with different professional backgrounds within the research group strengthened the credibility of this study [23].

### Ethical considerations

The study was approved by the Regional Ethical Review Board, Northern Sweden, in May 2016, decision No. 2016-242-32M. The autonomy, anonymity and informed written and oral consent of the participants were respected.

## Results

The theme that emerged from the experiences of women living with hyperhidrosis was Constantly guarding the female image. In order to be included in the female norm, women described struggling to keep up a feminine appearance while coping with the difficulties created by hyperhidrosis. Since hyperhidrosis was considered unhygienic, women used loose or neutral-shaped attire, which they associated with a feeling of having a less feminine body image. Moreover, this feeling reduced their self-esteem. Negative remarks and deficient knowledge about hyperhidrosis in the women’s proximity made the women feel poorly understood for the problems of having hyperhidrosis. Misunderstanding in close or intimate situations was described when women needed space to reduce the sweat production. Various strategies which would conceal the sweat had to be adopted to avoid withdrawing attention. Living with the impairments of hyperhidrosis also had an effect on choices regarding studies and career opportunities. Treatment with botulinum toxin was described as liberating and opened up their life again by removing the time and energy spent on preparations for meeting the daily life problems caused by excessive sweating. Categories and related subcategories are summarised in Table 3, and these are presented with illustrative quotes from interviews following below.

**Table 3:** Theme, categories and subcategories for women with primary hyperhidrosis.

Theme	Categories	Subcategories
Constantly guarding the female image	Misfitting the feminine norms	Fight to keep up a feminine appearance
		Overcome the awkwardness of intimacy on one’s own terms
	Avoiding the attention of others	Be singled out and made fun of by other women
		Feel misunderstood and questioned by others
	Passing like any woman	Ensure distance and staying ahead
		Receive life-changing treatment

### Misfitting the feminine norms

This category highlights the experiences participants made of their excessive sweating in relation to the societal norms of femininity, which also included the challenges in intimate relations. The subcategories *Fight to keep up a feminine appearance* and *Overcome intimacy on one’s conditions* cover aspects of not always fitting into the norms and expectations of female appearance because of the excessive sweating.

#### Fight to keep up a feminine appearance

Women described how, while society tended to enforce problematic aspects such as being hairy or sweaty as unfeminine, they tried to adapt to these expectations by concealing their excessive sweat. Being attractive or good-looking was described as an important feminine attribute, which had to be maintained. Sweating was in general considered unhygienic and reflecting badly on individual character, which women associated with being non-feminine. A consequence of having hyperhidrosis was that they found it difficult to buy attire specifically made for women, such as dresses, or tight or colourful clothes, since these would more easily reveal their excessive sweating. Instead, they had to rely on attire that was dark or white, baggy and more practical, in camouflaging the sweat marks. Also, women refrained from borrowing attire from friends or trying out clothes in the dressing room with their female friends, since they could risk leaving sweat marks and reveal their excessive sweat problems. Therefore, social gatherings with other female friends were sometimes avoided to not expose hyperhidrosis. Women expressed they had to control their body to keep a desired appearance, and it was important to hide the hyperhidrosis. Not being able to control the sweating and leaving marks on items had a negative effect on self-esteem and the self-image of the women, making them feel inferior to other women without hyperhidrosis.

*It is embarrassing and humiliating to be told about the sweating*. *Others feel it’s uncomfortable*, *disgusting and unclean*. *A lot focuses on controlling the body*, *and sweating is not a feminine thing*. *It’s maybe ok if you are a man*. *I don’t know*.

Participant #11

#### Overcome the awkwardness of intimacy on one’s own terms

The excessive sweating caused by hyperhidrosis was described as most problematic in intimate situations at the beginning of a relationship. The sweat could make the skin get stuck on clothes or sheets, or unintentionally on the body parts of their partner. Not being able to stroke the partner’s skin gently with fingers or the palms, due to wet hands, was a challenge in intimate situations. Loss of focus during intimacy could occur, and the women described an urge to have a free space to cool down, to reduce the sweat production. However, such distancing would send wrong signals, as if the intimacy was not desired. A sense of guilt and a feeling of not being attractive in intimate relationships were described as insuperable. Some women described how they had found solutions for intimate situations such as avoiding hugging above the shoulders so that their sweaty armpits were not exposed or taking a cold shower before sex to reduce or remove the sweat. While different measures helped for a short while, their strategies were successful enough to get them through the early stages of the relationship. An interesting remark was that women who had been in a stable relationship for many years still felt hesitations and concerns about how their partner’s perception of them being intimate or having sex was affected by the hyperhidrosis.

*The thought of being intimate with someone except my partner*, *who doesn’t know about my excessive sweating*, *is really worrisome*. *Getting stuck to sheets and being soaking wet during sex feels unclean*.

Participant #7

### Avoiding the attention of others

Women with hyperhidrosis not only struggled with their female appearance, but they also revealed that their condition was misunderstood by others and even close family members. Receiving negative comments or being made fun of by other women contributed to further efforts to hide and to minimise unwanted attention. The subcategories *Feel misunderstood and questioned by others* and *Be singled out and made fun of by other women* further describes these aspects of the experiences.

#### Feel misunderstood and questioned by others

Family members, partners and friends were described as important in confirming and accepting the problems experienced by women from hyperhidrosis. It was described that the consequences of hyperhidrosis in their daily life could not be understood by others in their proximity. While the increased production of sweat was understandable, others failed to apprehend the time and energy the women put into concealing the sweat, and the stress and anxiety these measures created. Therefore, disclosing the excessive sweating created further shame or a sense of needing to apologise for bringing the issue forward for discussion. Friends, family members and healthcare professionals could also question the severity of hyperhidrosis or offer their own explanations of what might cause the excessive sweating, which often were not helpful. Women felt they were struggling alone with their concerns, whether excessive sweating was physiological and present one day, or caused by a disease and permanent. Deficient knowledge about hyperhidrosis among family members, friends and society in general was described as a probable reason for the deficient understanding. Trust, and a context in which they did not felt humiliated discussing hyperhidrosis, were suggested as important for gaining understanding. Women described that being understood reduced the anxiety and time and energy spent on concealing hyperhidrosis.

*I never understood that my excessive sweating was actually a disease*. *I could speak to my parents but then they told me don’t worry*, *we will just buy you new clothes*. *They never understood the extent of my problems*.

Participant #5

#### Be singled out and made fun of by other women

Women had experienced being laughed at or receiving negative comments about having excessive sweating. It was usually other women telling them how they should behave or act which was considered painful and not constructive. These remarks led women to avoid wearing desired attire or going to the gym or other social gatherings. Several participants described how they were told by family members to wash their hands, when learning to knit at a young age. It did not help, since the yarn still became soaked by sweat, but it made them stop knitting so as not to be reminded of their hyperhidrosis. As others would judge their condition instead of their competence, the participants avoided certain work-related tasks or occupations. Having a job that required giving presentations or lectures was considered too uncomfortable, since they could be subjected to the prejudices of the audience if the sweating was noticed. The risk of getting negative feedback about their excessive sweating, in combination with having been negatively commented on earlier in life, influenced their choices regarding education or career ambitions.

*I was being helped by my mother with knitting when she told me to wash the sweat from my hands first*. *Being reminded of it wasn’t easy*. *I wanted to tell her I know this*, *you don’t have to tell me since it doesn’t help*. *I am already tormented by it*.

Participant #6

### Passing like any woman

To pass like other women and fit with societal expectations of females, all participants described having various strategies to cope with the excessive sweating. The botulinum toxin treatment was perceived as life-changing and enabled them to appear like other females The experiences of women regarding these aspects are described in the following subcategories: *Ensure distance and staying ahead* and *Receive life-changing treatment*.

#### Ensure distance and staying ahead

Social distancing and avoiding tasks that made them the centre of attention from others was described as reducing the risk of a sweat attack. Trying to decrease the temperature by opening a window at work or not having the radiators on were also helpful strategies. Women who could not adapt their environment wore paradoxically thicker clothes, pads or toilet paper in their armpits, which increased sweat production but gave them time to act if the sweating became noticeable. Some women described using male deodorants in their armpits, as they assumed them to be more effective in reducing the sweat production. Greeting and handshaking was most problematic for women with palmar hyperhidrosis, who used only some fingers or a partially closed palm in greeting others. Having sets of the same colour and material of clothes, keeping extra clothes at work and having clothes in reserve on a trip relieved the stress and anxiety of not being able to conceal hyperhidrosis. In worst-case scenarios and on a bad day, taking sick leave from work was the last option.

*I try to avoid things exacerbating my sweat problems*. *It has happened that I have taken sick leave to avoid exposing myself to specific events or gatherings*.

Participant #10

#### Receive life-changing treatment

Treatment with botulinum toxin stopped excessive sweating and contributed to a positive change in the women’s life. They no longer needed to conceal the sweat marks or abstain from disclosure, since the excessive sweating was not present. Anxiety and stress related to hyperhidrosis in general and regarding specific events, for example, during the summer when sweating could be more pronounced and tormenting or while having to wear work uniforms, was not a concern anymore. Sporting activities which had been avoided because of hyperhidrosis affecting the grip ability or physical or visual functions were performed without any problems. Feeling one’s skin as warm instead of wet and cold was described as positive, with intense emotions revealed by some. Women also described daily social interactions such as shaking hands, writing on a paper or wearing any clothes they wanted could be performed without complications after treatment. Being able to function in interactions with others without suffering from the negative effects of hyperhidrosis, and no longer having to think about the quiet handicap, was described by women as liberating and life changing.

*It is very impolite to not shake hands in my work*. *It would be a social handicap*. *I can now after treatment shake hands without being ashamed*. *It is an enormous liberation*.

Participant #8

## Discussion

This is the first study that exclusively investigated the experiences of women living with primary hyperhidrosis. The main theme that emerged was *Constantly guarding the female image*. The theme illustrated how hyperhidrosis made women feel less feminine, which also made them avoid attention, and how they tried to shield themselves from being made fun of. Different strategies were applied to protect themselves from revealing their condition and to pass like any woman. Botulinum toxin was regarded as a life-changing treatment and liberating the women from the burdens of hyperhidrosis.

In a previous study, we presented men’s experiences of living with hyperhidrosis and found that although hyperhidrosis was associated with being unclean and captive to its burdens, men didn’t report the disease disrupting the male image [10]. In this regard, women’s experiences of hyperhidrosis differ from men’s, as they considered hyperhidrosis unfeminine and unattractive. The experiences of having a chronic condition are influenced by norms of masculinity and femininity [24]. Women tend to be socialised as selfless and attentive to the need of others, and to construct their illnesses as physically troubling [25, 26]. For men, rather than the need to maintain an appearance, the body is socialised as a tool for achieving abilities, and masculinity is associated with autonomy, competitiveness and control [27]. Thus, there are reasons to assume that gendered norms and societal expectations may contribute to women’s perceptions of living with hyperhidrosis, as described in this study.

The aspect of femininity, described by women with hyperhidrosis, could provide an explanation to the discrepancy between women and men regarding perceptions of health and, importantly, help-seeking behaviours. Gender aspects in experiences of a disease and how they are expressed are important since they could also affect the care being provided [28]. For example, by comparing sex regarding obesity and bariatric surgery, it has been reported that while women are not overrepresented suffering from obesity, they undergo bariatric surgery much more often than men [29]. Reasons such as social and cultural norms have been described as important factors in shaping women’s perceptions of the ideal body image [30]. In hyperhidrosis, these gendered aspects could potentially have an impact on subjective scales and self-assessments by the patients in clinical settings. Considering that objective methods for measuring sweat production are seldom used during assessments of patients with hyperhidrosis, healthcare professionals need to be aware that women suffering from hyperhidrosis could present their impairments differently from men. This could result in an unequal care as women are more often treated due to probable reasons such as social expectations instead of severity and the impairments of hyperhidrosis.

Another interesting finding was that excessive sweating in women was exclusively commented on by other women. Although there is no research regarding women evaluating each other’s bodies in hyperhidrosis, it has been reported that women experience a discrepancy between their own body and the ideal female body [30]. As suggested, the pressure to conform to the body ideal is higher in women, since in media and western societies beauty is strongly associated with femininity [31]. For example, women tend to engage more in talk about weight and to talk in groups about their body dissatisfaction [32]. Changing norms is a difficult process that requires time and resources. Norms regarding body fluids are often associated with an individual’s preference for personal hygiene and are deeply rooted within western societies. Increasing awareness and knowledge of unknown diseases such as hyperhidrosis could create more understanding about the problems those affected face in life. Clinicians educating female patients with hyperhidrosis could encourage them to become ambassadors for the change they would like to see in society. This is a resource-efficient way to spread knowledge and to facilitate for others suffering from the disease to get confirmation and help for their problems. Providing accurate and available information for the public from healthcare services is another method that could increase the possibility of obtaining medical help and make women and the people close to them aware of hyperhidrosis. It is also important that both researchers and clinicians increase awareness regarding hyperhidrosis by engaging in discussions and providing relevant information through media. In addition, supporting organised patient groups with available research could empower patients with hyperhidrosis to act as a bridge between policymakers, academia and industry and become drivers of progress.

Finally, women in this study considered botulinum toxin treatment as life-changing and liberating. Although the positive gains of botulinum toxin treatment in hyperhidrosis may not be controversial for clinicians and patients, Wade et. al [33] described moderate-quality evidence supporting botulinum toxin treatment for axillary hyperhidrosis and low-quality evidence for the treatment of palmar hyperhidrosis. The results by Wade et al. were confirmed in a recent study by Stuart et al. [34]. Too few randomised trials, often of poor quality, was described affecting the level of evidence [33, 34]. Thus, great care is necessary when comparing our results with these reviews as data from qualitative content analysis is neither quantifiable nor represents a weighted average of a systematic review. While the aim of our study not was to investigate the effect of botulinum toxin we cannot exclude that those who abstained participation may have experienced botulinum toxin as less effective. We do not find a conflict with the mentioned reviews since heterogenicity in systematic reviews my lead to estimates which are not representative in individual studies. Therefore, further research is warranted to evaluate the efficacy and safety of botulinum toxin treatment in hyperhidrosis.

### Strengths and limitations

While qualitative content analysis is a method subject to individual interpretations creating challenges to results and conclusions yielded, having several researchers with different backgrounds and fields of expertise strengthens the findings, subjecting them to different aspects as done in this study. It is also important to mention the ease and depth that women showed when sharing their positive and negative experiences of hyperhidrosis, although being interviewed by a male researcher working in healthcare. Women also appeared to be comfortable in disclosing their sexuality-related experiences during the interviews, as they provided rich descriptions of how hyperhidrosis interfered in their intimate parts of life, which contributes to the strength of this study.

While a limitation of qualitative research is the ability to transfer the findings to a wider population, as social and cultural contexts are important to consider, the findings in this study could give insight into how women with hyperhidrosis describe the problems they experience. Further, predominantly talkative participants may have volunteered to take part; thus, the experiences may reflect the thoughts of more open and talkative individuals. The findings will improve healthcare professionals’ knowledge to enable a better understanding and ability to identify and assess the obstacles women with hyperhidrosis face in their everyday life and provide tailored treatment options.

## Conclusions

Primary hyperhidrosis in women disrupts the feminine appearance, lowers self-esteem and hinders social interactions. Clinicians need to be aware of gender aspects influencing the experiences described by women suffering from hyperhidrosis or risk missing in patient assessments important disabilities that excessive sweating creates. Educating patients, providing accurate information regarding the disease and cooperating with patient groups are important for increasing awareness and achieving progress in care for women with hyperhidrosis. Further research is needed for developing tools for clinicians in objective assessments in patients with hyperhidrosis.

## Supporting information

S1 Protocol Interview guide English. (PDF)

S2 Protocol Interview guide Swedish. (PDF)
