# Supplementary material for: ‘Excessive sweating is not a feminine thing’: A qualitative study of women’s experiences suffering from primary hyperhidrosis
Source: PLoS One. 2021 Jul 15;16(7):e0254689. doi: 10.1371/journal.pone.0254689 (PMC8282083; doi:10.1371/journal.pone.0254689)
Supplement: S1 Protocol — (PDF) [file pone.0254689.s001.pdf]

Interview guide

* Information given about the study and the interview procedure ☐

Code………. Date………

Sex………. Age……. Occupation………

Onset of the disease: ……years

Relationship:…………

Topics to discuss during the interviews

1. Start with **experiences of living with** primary hyperhidrosis
   - -Could you describe and provide example of situations…
   - -What's your thought about it?
   - -Affecting family, friends and relatives

2. Onset of symptoms or problems associated with hyperhidrosis
How it affected the overall health
Adolescents
Adulthood
   - Occupational life (Sick leave? Performance?)
   - Social life (In different situations and with others)
   - Private life (Relation to partner(s), sex och living together)
   - Leisure's (sports, hobbies)

3. Turning point
4. Coping mechanisms
6. What is culturally accepted concerning hyperhidrosis

7. What is health for you? What is being unhealthy?
   - -Could you describe a situation or a day when you experienced being healthy or unhealthy?

9. Conclusion. Have we forgotten anything?
