# Supplementary material for: ‘Excessive sweating is not a feminine thing’: A qualitative study of women’s experiences suffering from primary hyperhidrosis
Source: PLoS One. 2021 Jul 15;16(7):e0254689. doi: 10.1371/journal.pone.0254689 (PMC8282083; doi:10.1371/journal.pone.0254689)
Supplement: S2 Protocol — (PDF) [file pone.0254689.s002.pdf]

Intervjuguide

* Informera om studien och vad som ska hända under intervjun ☐

Kod:………. Datum………

Kön:………. Ålder:……. Sysselsättning:………

Sjukdom sedan: ……år

Levnadsförhållande:…………

Områden att diskutera under intervjun

1. Börja med **upplevelser och erfarenhet** om primär hyperhidros
   - -Kan du beskriva och ge exempel av situationer…
   - -Vad **tänker du** om det?
   - -Påverkar familj, vänner och släkt

2. Debut av symtom och besvär kopplat till hyperhidros.
Hur sjukdomen påverkade den allmänna hälsan
Tonårslivet
Vuxenlivet
   - Arbetsliv (sjukskrivning? Arbetsförmåga?)
   - Socialt liv (I olika situationer och med andra)
   - Privatliv (Relation till partner(s), sex och samlevnad)
   - Fritid (idrott, hobbys)

3. Vändpunkt?
4. Coping mekanismer?
6. Vad anser du är kulturellt accepterad gällande hyperhidros

7. Vad är hälsa för dig? Vad är ohälsa?
   - -Kan du berätta om ett tillfälle eller en dag när du upplevde hälsa eller när du upplevde ohälsa?

9. **Sammanfatta**, något vi har **glömt**?
